# Uterine Fibroids: From Molecular Oncology to Reproduction

**DOI:** 10.1155/2018/6284875

**Published:** 2018-09-03

**Authors:** Andrea Tinelli, William H. Catherino, Antonio R. Gargiulo, Brad S. Hurst, Ospan A. Mynbaev, Daniele Vergara, Antonio Giuseppe Naccarato

**Affiliations:** ^1^Department of Gynecology and Obstetrics, Division of Experimental Endoscopic Surgery, Imaging, Minimally Invasive Therapy and Technology, Vito Fazzi Hospital, Lecce, Italy; ^2^Laboratory of Human Physiology, Phystech BioMed School, Faculty of Biological & Medical Physics, Moscow Institute of Physics and Technology (State University), Dolgoprudny, Moscow Region, Russia; ^3^Department of Obstetrics and Gynecology, Uniformed Services, University of the Health Sciences, Bethesda, MD, USA; ^4^Program in Reproductive Endocrinology and Gynecology, Eunice Kennedy Shriver National Institute of Child Health and Human Development, NIH, Bethesda, MD, USA; ^5^Center for Infertility and Reproductive Surgery, Brigham and Women's Hospital, Boston, MA, USA; ^6^Division of Reproductive Endocrinology and Infertility, Department of Obstetrics and Gynecology, and Dickson Advanced Analytics, Carolinas Medical Center, Charlotte, NC, USA; ^7^Moscow Institute of Physics & Technology (State University), Russia; ^8^The Pirogov Russian National Research Medical University, Russia; ^9^The INP of The Russian Academy of Sciences, Russia; ^10^Department of Biological and Environmental Sciences and Technologies, University of Salento and Laboratory of Clinical Proteomic, “Giovanni Paolo II” Hospital, ASL-Lecce, Italy; ^11^Department of Translational Research and New Technologies in Medicine and Surgery, University of Pisa, Italy

Smooth muscle uterine tumors include a large group of neoplasms representing the entire spectrum, from benign to malignant cancers. This includes benign fibroids, STUMPs (smooth muscle tumors of uncertain malignant potential), and leiomyosarcomas (LMS). Uterine fibroids, also known as myomas or leiomyomas, are the most common benign tumors of the genital organs of women of childbearing age. Literature data show that up to 77% of women have fibroids, either depending on the study population or by diagnostic techniques applied to myoma detecting.

Symptomatic fibroids account for approximately over 200,000 hysterectomies and 50,000 myomectomies annually in the United States and may cause significant morbidity, including abnormal uterine bleeding, infertility, or bulk symptoms causing pelvic discomfort. Fibroids have a major impact on fertility, with an overall significant adverse effect for fibroids on implantation rate and spontaneous abortion rates when compared with infertile women without fibroids. Impaired peristalsis, inflammatory response and physical deformity of the uterine cavity by fibroids may all play an overall role in reducing fertility outcomes. Moreover, fibroids are associated with abortion and adverse obstetric outcomes, abnormal placentation, placental abruption, premature rupture of membranes, postpartum hemorrhage, and preterm birth. Fibroids are more common in women over 40 years, when fibroids and infertility are more frequent and prevalent, which increases the risk of this association and of associated complications.

Because of the wide-spread nature of this disease, uterine fibroids are the number one pathologic cause of surgery in gynecological patients. In addition, this common female pathology has a negative impact on wellbeing, with a significant female morbidity and impairment of quality of life. According to the literature, 40-60% of all hysterectomies are scheduled for symptomatic fibroids, as the most common worldwide indication.

Fibroids consist mainly of smooth muscle cells with different amounts of fibrous tissue surrounded by a neurofibrovascular network, the myoma pseudocapsule, which enables their enucleation and enhances myometrial healing. Because of the wide-spread nature of this disease, clinical interventions and costs associated with uterine myomas are constantly growing. For this reason, the interest and research in different myoma aspects including transcription factors and gene targets involved in myoma development and new pharmacological and surgical treatments have grown exponentially in the last several years.

Myomectomy is not a risk-free operation, since the surgical procedure can cause mechanical infertility and can be associated with infection, injury to adjacent tissues, and hemorrhage. There are robust surgical outcome data supporting the use of a minimally invasive surgery (MIS) such as laparoscopy and hysteroscopy over laparotomy. Perioperative outcomes and return to normal activity are significantly better with MIS.

Differentiating between leiomyoma and leiomyosarcoma presurgically can be difficult, particularly in premenopausal patients. Frequently, myoma at rapid growth can be confused and misdiagnosed with a LMS in women over 40 years of age. This malign neoplasm is a rare, aggressive cancer of the uterine muscle cells, clinically difficult to distinguish from benign leiomyomas. The annual incidence of leiomyosarcoma (LMS) is less than two women per 100 000 based on the population-based Surveillance, Epidemiology and End Results (SEER) database from the USA National Cancer Institute, so it is a rare tumor (3 to 7 per 100,000 in the USA population). The incidence of LMS increases with age and is diagnosed usually after menopause, around 60 years of age. It is more common in the African American race and after prolonged use of tamoxifen of over 5 years. LMS has a poor prognosis even in early-stage disease due to an early hematogenous spread. Pritts and colleagues estimated in 2015 the likelihood of finding a leiomyosarcoma in general population. The estimated rate of leiomyosarcoma was 0.51 per 1000 procedures or approximately one in 2000. In assessment by meta-analysis, there was a substantially lower estimate of 0.12 leiomyosarcomas per 1000 procedures or approximately one leiomyosarcoma per 8300 surgeries. Literature recently reviewed the risk of occult leiomyosarcomas found at surgery for presumed benign fibroids, since leiomyosarcomas are most commonly diagnosed following myomectomy or hysterectomy for presumed leiomyomas. Moreover, there is no pelvic imaging modality that can reliably differentiate between benign leiomyomas and LMS. In addition, there are currently no validated clinical or radiographic criteria to differentiate a leiomyoma from a LMS, as the final diagnosis made histopathologically after hysterectomy or myomectomy.

Our special issue, which had opened for 3 months in the end of 2017, focused on uterine fibroids, focusing on their impact on female wellbeing and treatment. An article of H. S. Saleh et al. investigated the impact of fibroid on obstetric outcome in pregnancy, by a prospective observational study in Egypt, on 64 pregnant patients with >2 cm fibroids. Patients were followed during antenatal period clinically and scanned by ultrasound, which was performed at starting of pregnancy and during subsequent visits, to assess the change in the size of the fibroid and other obstetric complications. Authors recorded increased size of fibroids, complications during pregnancy, labor, delivery, and changes in mode of delivery. They concluded that while most fibroids in pregnancy are asymptomatic, pregnant patients who have fibroids have a higher incidence of complications throughout antepartum, intrapartum, and postpartum period. Therefore, they should be carefully screened in the antenatal period through regular follow-up.

Another study of A. Tinelli et al. investigated the difference of myoma pesudocapsule thickness measured in 200 submucous, intramural, and subserous fibroids by histology and ultrasound and evaluated its possible correlation with fertility impairment caused by fibroids. The thickness of the pseudocapsule was greater for the submucosal myomas, compared, respectively, to the intramural and subserous, suggesting a potential role in fertility or in myometrial healing.

The investigation of I. Mazzon et al. analyzed which variables influenced the completion of a cold loop hysteroscopic myomectomy in a one-step procedure, by a retrospective cohort study of 1434 operations consecutively performed and 1690 removed fibroids. The multivariate analysis showed that the size, the fibroids' number, and the age of patients were significantly correlated to the risk of a multiple-step procedure. No correlation was revealed with the fibroid grade, parity, and the use of presurgical GnRH-agonist therapy. Authors concluded that in case of multiple fibroids the intramural development of submucous myomas did not influence the completion of cold loop hysteroscopic myomectomy in a one-step procedure. The size of myomas and the age of patients were significantly correlated with the need to complete the myomectomy in a multiple-step procedure.

J.-J. Li et al. reviewed the management of adenomyosis in women wishing to improve or preserve fertility, starting form adenomyosis pathogenesis, clinical presentation, diagnosis by instrumental and histological detection, and pharmacological and surgical therapy, evaluating the impact of patients' fertility.

Finally, T. D. Lewis and colleagues published a comprehensive review of the pharmacologic management of uterine leiomyoma, evaluating the effect of nonhormonal and hormonal treatments, aromatase inhibitors, gonadotropin-releasing hormone analogs, and selective progesterone receptor modulators on fibroids.

In conclusion, we expect that this special issue provides a valuable update on the scientific progress of uterine research, notably adding insight and future direction on scientific research and fibroid clinical practice.

*Andrea Tinelli* *William H. Catherino* *Antonio R. Gargiulo* *Brad S. Hurst* *Ospan A. Mynbaev* *Daniele Vergara* *Antonio Giuseppe Naccarato*

